# Advancing a Human Onchocerciasis Vaccine From Antigen Discovery to Efficacy Studies Against Natural Infection of Cattle With *Onchocerca ochengi*

**DOI:** 10.3389/fcimb.2022.869039

**Published:** 2022-04-04

**Authors:** Bin Zhan, Maria Elena Bottazzi, Peter J. Hotez, Sara Lustigman

**Affiliations:** ^1^ Texas Children’s Hospital Center for Vaccine Development, Baylor College of Medicine, Houston, TX, United States; ^2^ Laboratory of Molecular Parasitology, Lindsley F. Kimball Research Institute, New York Blood Center, New York, NY, United States

**Keywords:** *Onchocerca volvulus*, onchocerciasis, vaccine, animal model, clinical trial

## Abstract

Human onchocerciasis is a devastating neglected tropical disease caused by infection of the filarial nematode *Onchocerca volvulus*. The infection can cause irreversible visual impairment or blindness and stigmatizing dermatitis. More than 32 million people were estimated to be infected with *O. volvulus* in Africa, and 385,000 suffered from blindness. Even though the implementation of mass drug administration (MDA) with ivermectin has reduced the global prevalence of onchocerciasis, *O. volvulus* infection remains challenging to control because MDA with ivermectin cannot be implemented in endemic areas co-endemic with loiasis due to the risk of severe adverse events. There is also emerging drug resistance to ivermectin that further complicates the elimination of onchocerciasis. Thus, the development of a vaccine that would induce protective immunity and reduce infection burden is essential. Efforts to develop prophylactic and/or therapeutic vaccines for onchocerciasis have been explored since the late 1980s by many researchers and entities, and here we summarize the recent advances made in the development of vaccines against the infection of *O. volvulus* and onchocerciasis.

## Introduction

Human onchocerciasis or river blindness is a serious parasitic disease caused by infection of the filarial nematode *Onchocerca volvulus* transmitted through the bite of an infected blackfly (Lustigman, 2018). It is a devastating neglected tropical disease with an adverse impact on human health and socioeconomic status because it causes irreversible visual impairment or blindness and stigmatizing dermatitis (Schwartz et al., 2020). Some evidence indicates that onchocerciasis may also cause seizures and epilepsy (Romo, 2020). More than 32 million people were estimated to be infected with *O. volvulus*, most of whom live in sub-Saharan Africa, before the mass drug administration (MDA) with ivermectin was initiated in 1995 (Coffeng et al., 2013). Approximately 385,000 suffered from blindness, 944,000 from low vision, and 3 million from severe itching (WHO, 2010). Even though the implementation of MDA with ivermectin and vector control results in a significant reduction in the global prevalence of onchocerciasis, it remains highly prevalent. Recent estimates from the Global Burden of Disease Study 2019 indicate that 19.1 million people live with onchocerciasis with the highest prevalence and disease burdens in the Democratic Republic of the Congo and neighboring Central African Republic, South Sudan, Chad, and Cameroon (Institute for Health Metrics and Evaluation). In these endemic areas on the African continent, onchocerciasis results in an estimated 1.23 million years lost from disability due to debilitating effects of skin disease and vision impairment (G. B. D. Disease Injury Incidence and Prevalence Collaborators, 2018). In 2019, more than 200 million people in 30 countries required preventive therapy for onchocerciasis (WHO, 2020).

MDA using ivermectin alone may not be sufficient to achieve onchocerciasis elimination due to several key barriers. First, MDA with ivermectin cannot be implemented in areas co-endemic with onchocerciasis and loiasis due to the risk of severe adverse events (SAEs) including encephalopathy in patients infected with filarial *Loa loa* treated with ivermectin (Gardon et al., 1997; Kelly-Hope et al., 2014). Failure to control onchocerciasis in these regions allows the establishment of reservoirs of *Onchocerca* infection to spread into neighboring areas that previously received MDA (Kelly-Hope et al., 2014). Another concern for ivermectin MDA is the emergence and/or potential widespread of partial or complete ivermectin drug-resistant *O. volvulus*, which may reduce the long-term effectiveness of ivermectin when used alone in the endemic regions (Lustigman and McCarter, 2007; Turner et al., 2013). Based on these limitations, a disease modeling study concluded that it may not be possible to eliminate onchocerciasis even after 50 years of annual ivermectin MDA; this has prompted suggestions to adopt a biannual treatment program to reach the goal of the London Declaration on Neglected Tropical Diseases Goals for onchocerciasis (Dadzie et al., 2003; Turner et al., 2014).

Based on these limitations, the development of new tools such as new drugs and vaccines will be necessary to ensure sustainable onchocerciasis elimination (Hotez et al., 2015; Lustigman, 2018), as proposed in the WHO, 2021–2030 Roadmap on Neglected Tropical Diseases (WHO, 2021). The development of prophylactic and therapeutic vaccines will complement current and future chemotherapeutic programs and thus accelerate the elimination efforts of onchocerciasis worldwide (Hotez et al., 2015; Makepeace et al., 2015). The efforts to develop prophylactic and/or therapeutic vaccines for onchocerciasis have been explored since the late 1980s by many researchers and entities, and here we summarize the recent advances made in the development of vaccines against the infection *of O. volvulus* and onchocerciasis.

## Animal Models

A major obstacle for vaccine development against *O. volvulus* infection was the lack of a suitable animal model used to evaluate the immunogenicity and vaccine efficacy of human vaccines. The *O. volvulus* parasite only naturally infects human and gorillas and has rarely been observed in other primates (Vandenberghe et al., 1964; Duke, 1980; Abraham et al., 2002). Even though some primates were able to be experimentally infected with *O. volvulus* and in which adult filarial worms can develop (Eberhard et al., 1991; Eberhard et al., 1995), it is not feasible or practical to use non-human primates as an animal model on which to test a vaccine against onchocerciasis at the early stage due to high cost, logistical limitations, and ethical constraints (Abraham et al., 2002; Lustigman et al., 2002; Abraham et al., 2021). Another limitation for vaccine development against onchocerciasis is the difficulty to obtain the third-stage infective larvae (L3) without definitive animal hosts infected with the parasite to provide the microfilariae needed to infect blackflies. Infected blackflies by feeding on consented infected individuals are the only source of infective L3. The success in the cryopreservation of infective larvae of *O. volvulus* greatly facilitated the ability to conduct challenge studies in the laboratory (Trpis et al., 1993) using the following models.

### Mouse Diffusion Chamber Model With *O. volvulus*

Even though human *O. volvulus* cannot establish infection in rodents, a mouse model was successfully established for evaluating the protective immunity to the larvae of *O. volvulus* within diffusion chambers implanted under the skin of mice (Lange et al., 1993). The chamber model allows the humoral and cellular immune components to go through the chamber freely for observation of their effects on L3 in the microenvironment. This chamber model has been successfully used to evaluate the protective immunity induced by the immunization of irradiated L3 and other recombinant vaccine candidates (Lange et al., 1993; Lange et al., 1994). The mouse model with the implanted chamber was selected as a moderate throughput means to screen for the vaccine candidates (Hess et al., 2014). However, the limitation of the mouse chamber model is that the challenged *O. volvulus* L3 only live for a short time and do not develop into adult worms or produce microfilaria, and thus it is not suitable for testing therapeutic vaccines (Abraham et al., 2002).

### Cattle Model With *O. ochengi*

*O. ochengi* is a species of *Onchocerca* that naturally infects cattle. It is closely related to human *O. volvulus* and shares extensive molecule similarity and antigen cross-reactivity (Hoch et al., 1992). Both species are endemic in the same areas and transmitted by the same black fly vector *Simulium damnosum*. An epidemiological study showed that humans acquired cross-protection against *O. volvulus* infection by exposure to infected larvae of *O. ochengi* in the same endemic region (Wahl et al., 1994; Wahl et al., 1998). There is evidence for naturally acquired protective immunity against *O. ochengi* infection in cattle, similar to those individuals with putative resistance to *O. volvulus* (Wahl et al., 1998). These putatively immune cattle were significantly less susceptible to heavy field challenge than were naïve control cattle (Tchakoute et al., 2006). Experimental infection with *O. ochengi* demonstrated similar kinetics of an immune response as those reported in *O. volvulus* infection (Trees et al., 2000). A serological investigation in cattle for recombinant proteins of *O. volvulus* revealed a high degree of immunological cross-reactivity between the antigens of *O. volvulus* and *O. ochengi* (Graham et al., 1999). These studies demonstrate the similarity in the biological and immunological properties between *O. ochengi* in cattle and *O. volvulus* in humans. Such findings suggest that the naturally infected cattle/*O. ochengi* model can be used to test prophylactic or therapeutic vaccines and/or drugs under experimental and field conditions (Trees et al., 2000; Tchakoute et al., 2006; Makepeace and Tanya, 2016). This bovine model of *O. ochengi* has been successfully used to test therapeutic drugs and protective immunity of onchocerciasis by the WHO Drug Development Research Macrofil program and the Edna McConnell Clark Foundation vaccine development program in experimental infections in Liverpool and field infections in northern Cameroon (Trees et al., 2000; Tchakoute et al., 2006; Makepeace et al., 2009; Lustigman et al., 2017; Bah et al., 2021).

### *Brugia malayi*-Gerbil Model

Since *B. malayi* and *O. volvulus* are two genetically related filariae that share a similar life cycle and antigenicity, the *B. malayi*-gerbil model can be used to reflect the efficacy of the onchocerciasis vaccine (Morris et al., 2013; Arumugam et al., 2016). Gerbils are permissive to the infection of human filarial *B. malayi* by subcutaneous or peritoneal injection of L3. The adult worms can be developed in the spermatic cord and lymphatic vessels, and microfilariae appear in blood 79 to 116 days postinfection and last for at least 26 weeks (Ash and Riley, 1970; Morris et al., 2013). Vaccination of gerbils with irradiated *B. malayi* L3 induced a 56%–91% reduction in recovered worms and a complete protection against microfilaremia (Yates and Higashi, 1985). This is a potential backup model to test the vaccine efficacy of the homologues of *O. volvulus* vaccine candidates for preventive, therapeutic, or even transmission-blocking vaccines (Abraham et al., 2021).

## Acquired Immunity Against *O. volvulus* Infection in Humans

There is strong epidemiological evidence showing that acquired immunity against *O. volvulus* infection occurs in some people living in endemic areas. The population in hyperendemic regions with age >40 had a trend of decreased skin microfilariae (mf) density (Duke and Moore, 1968) or reduced number of adult worm-caused skin nodules (Albiez et al., 1984), indicating that chronic infections with *O. volvulus* may have developed a means of limiting further infections, although some of the individuals could be less susceptible to worm infection irrelevant to adaptive immunity. About 1%–5% of the population in hyperendemic areas do not exhibit patent infection or any clinical symptoms after living in endemic areas for extended periods of time and keeping constant exposure to the infection (Gallin et al., 1988; MacDonald et al., 2002). These individuals are believed to acquire immunity against infection of *O. volvulus* referred to as putatively immune (PI) (Elson et al., 1994). Another indication of acquired immunity is concomitant immunity, induced by the current infection to prevent newly infective L3 from developing into adult worms in order to keep a stable adult worm burden (Mitchell, 1990; MacDonald et al., 2002). This immunity is directed not against the concurrently infected adult worms or the microfilaria in infected subjects but against the reinfection with new L3. The acquired immunity was also observed in cattle. The cattle raised in endemic areas without detectable parasites were significantly less susceptible to the challenge with *O. ochengi* than age-matched naïve cattle (Tchakoute et al., 2006).

Understanding the immunological mechanisms underlying the protective immunity in PI subjects can lead to the identification of antigens involved in the protective immunity as vaccine targets against this infection. Immune sera collected from PI people specifically recognized many antigens in L3, L4, or microfilaria compared to sera from infected people, indicating that PI develop unique antibody responses that may be related to the protective immunity (Irvine et al., 1997). Previous studies have shown some associations between immunity and defined antibody and/or cellular responses in exposed individuals. Infection with *O. volvulus* induced strong IgG and subtype IgG responses to a 20-kDa antigen (GP20) in *O. volvulus*, especially with a high level of IgG3 in putatively immune persons, which indicates its importance in acquired immunity to onchocerciasis (Boyer et al., 1991; Stewart et al., 1995). Cellular response measurement demonstrated that PBMCs collected from PI individuals produced enhanced levels of IL-5 (Th-2), INF-ɣ (Th-1), and a higher level of GM-CSF responses than the infected group against *O. volvulus* larval/adult antigens, indicating that those PI have distinct antigen-specific cytokine responses (Turaga et al., 2000). PI individuals showed significantly higher antigen-specific IL-2 (Ward et al., 1988) and INF-Υ (Elson et al., 1995), indicating that Th-1 like responses are associated with immunity to onchocerciasis in putatively immune persons; however, other researchers did not find the correlation of cellular response with protective immunity in PBMCs from PI subjects (Gallin et al., 1988). All results indicate that putative immunity is associated with mixed Th1/Th2 responses against *O. volvulus* larvae (Greene et al., 1985; Turaga et al., 2000; MacDonald et al., 2002).

**Figure 1:**
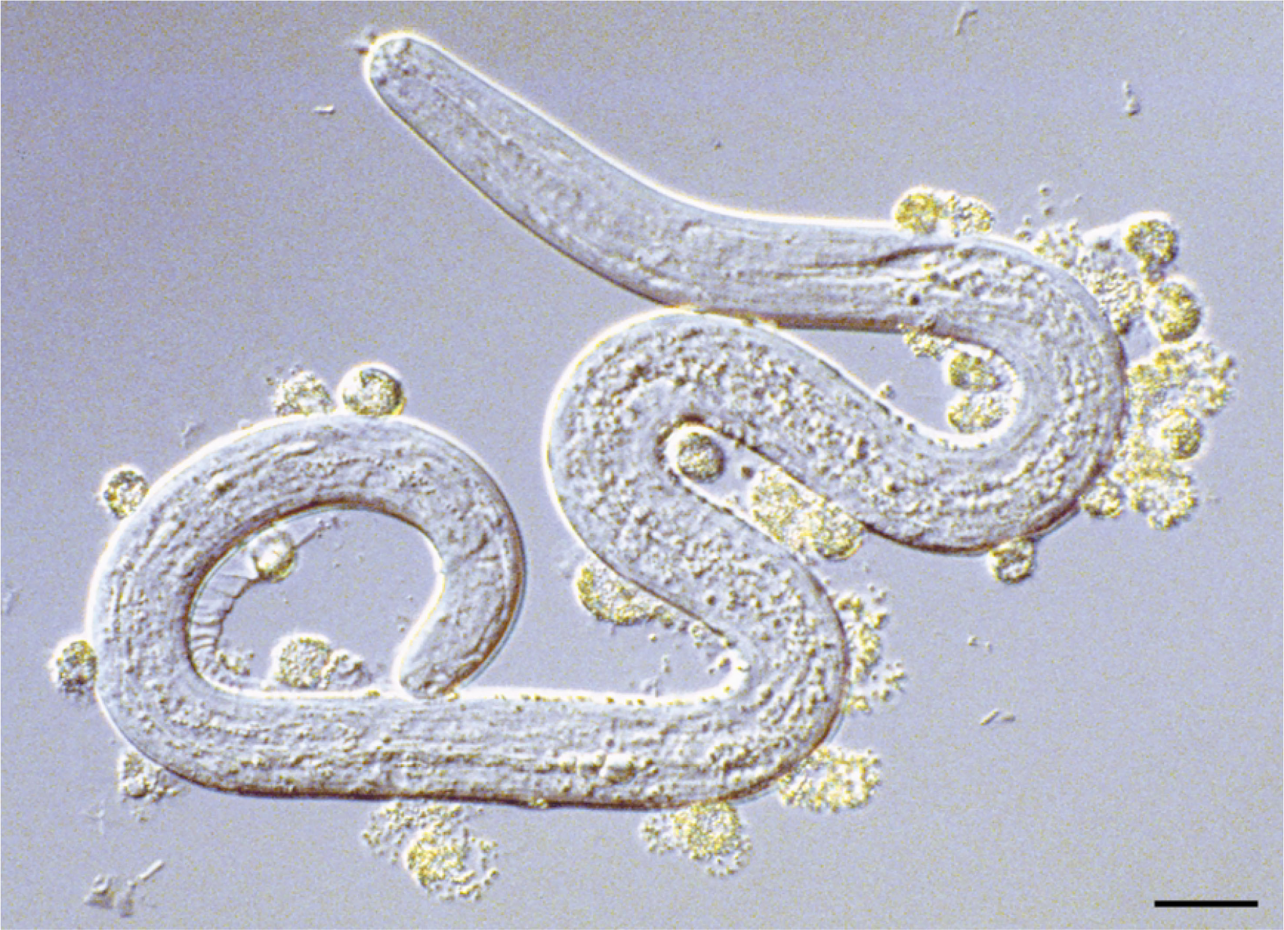
Granulocytes attaching and degranulating on the surface of larval *O. volvulus* recovered from a diffusion chamber, implanted in mouse immunized with irradiated *O. volvulus* third-stage larvae, reprinted from Abraham et al. (2002), with permission.

Another study observed that neutrophils or other immune effective cells (e.g., eosinophils) collected from endemic-infected or non-infected individuals (PI) adhered to infective L3 in the presence of specific IgG in their sera; it promoted the killing of L3 or inhibited the molting of L3 to fourth-stage larvae *in vitro* (**Figure 1**), indicating that this antibody-dependent cell-mediated cytotoxicity (ADCC) against L3 is involved in the immunological defense mechanism (Leke et al., 1989; Johnson et al., 1991; Johnson et al., 1994). These findings further suggest that specific protective immunity may occur in *O. volvulus* natural infection.

## Proof of Concept Using Irradiated Larvae

Infective larval worms that undergo attenuation through radiation, ultraviolet light, or other means have been used experimentally to investigate their vaccine efficacy and commercially to produce veterinary vaccines against canine ancylostomiasis (hookworm infection) (Steves et al., 1973) and bovine dictyocaulus lungworm infection (McKeand, 2000). UV-irradiated *Ascaris suum* eggs containing the infective larvae also induced significant protective immunity against a challenge infection (Urban and Tromba, 1984). They provide an early indication for the feasibility of larval antigens to induce protective immunity for human helminth infections, and this strategy was adopted as an initial approach for a human hookworm vaccine initiative (Schneider et al., 2011).

This approach was also used to determine if protective immunity can be induced by the irradiated L3 of *O. volvulus* at the early vaccine development stage. Mice immunized with a single dose of irradiated L3 of *O. volvulus* significantly reduced the survival of challenged *O. volvulus* L3 in implanted diffusion chambers; however, killed L3 could not induce the protective immunity indicating that irradiated L3 induces protective immunity that requires L3 to be alive, possibly related to the fact that living L3 can secrete proteins (Lange et al., 1993). Immunization induced by irradiated L3 induced high levels of antibody responses, including IgG1, IgG2a/2b, IgA, IgE, and IgM, which recognized *O. volvulus* L3 and adult antigens (Yutanawiboonchai et al., 1996). The killing of challenged larvae in immunized mice required direct contact with host immune cells, especially eosinophils. The protective immunity was associated with increased levels of IL-5, IL-4 (Lange et al., 1994), and IgE (Abraham et al., 2004), but not with INF-Υ response (Lange et al., 1994; Johnson et al., 1998), indicating that Th-2 response and eosinophil effector cells are important for the irradiated L3-induced protective immunity against a challenge infection with *O. volvulus*.

Cattle immunized with irradiated L3 of *O. ochengi* acquired sterile protection against experimental challenge and significant protection against natural infection in the field with a significantly reduced nodule load and microfilaria count as well. These results provide evidence of protective immunity induced by the irradiated L3 and the proof of principle for immunoprophylaxis under experimental challenge or field natural infection (Tchakoute et al., 2006). The proof of principle for vaccine efficacy against onchocerciasis in naturally exposed cattle has been also demonstrated against *O. lienalis* in cattle vaccinated with microfilaria extracts (Townson and Bianco, 1982).

Although significant or complete protection can be induced by the immunization of irradiated L3 against *O. volvulus* infection, the safety concern for the use of living larvae and the difficulties to obtain large quantity of infective L3 from infected blackflies has limited its application in human vaccine. However, the immune sera from animals immunized with irradiated L3 have been used to identify those L3 antigens potentially involved in the protective immunity and thus promising vaccine candidates. A similar approach was used to identify the first human hookworm vaccine candidate to enter phase 1 clinical trials, an antigen known as Na-ASP-2 (Bethony et al., 2008), before revising human hookworm vaccine candidates to focus on adult stage antigens (Adegnika et al., 2021).

## Roadmap to Identify Vaccine Candidates

Both prophylactic and therapeutic vaccines have been considered for control of onchocerciasis. An effective prophylactic vaccine should protect vulnerable populations living in endemic areas against *O. volvulus* new infections, especially for children 1–5 years old before being exposed to the infection. The therapeutic vaccine aims at reducing the adult worm burden and microfilariadermia that cause pathology. Both preventive and therapeutic vaccines could also contribute to lower transmission rates in the endemic region to complement and enhance the MDA with ivermectin endgame or reduce the use of ivermectin, thus potentially reducing the emergence of drug resistance (Lustigman et al., 2017).

A traditional roadmap to develop a vaccine against infectious diseases includes identifying vaccine candidates through immunological approaches to identify antigens recognized by the protective immune sera. Immunoscreening of a stage-specific cDNA library of *O. volvulus* with the sera from individuals who acquired protective immunity against infection after many years of exposure to natural infection in endemic areas or developed concomitant immunity (Lustigman et al., 2002) is the major approach to identifying the immunodominant antigens with potential as vaccine candidates. The antigens recognized by these protective sera could be the promising targets of prophylactic and/or therapeutic vaccines against *O. volvulus* infection (Abraham et al., 2021). The construction of cDNA libraries from critical life-cycle stages for the establishment of *O. volvulus* infection including infective L3 and the molting L3 (mL3), or microfilariae (Mf), has provided an important platform to screen for such vaccine targets.

However, some protective antigens with essential functions to maintain the life cycle of parasites may not be exposed to the host immune system during natural infection, such as digestive proteases that coat on the intestinal tract of the target parasite. These “hidden antigens” with essential functions cannot be identified using conventional immunological approaches (Hotez et al., 2016). In addition, parasitic helminths secrete a lot of proteins with immunomodulatory functions to reduce host immune response as a survival strategy to evade host immune attack. These immunomodulatory proteins may not induce a strong immune response during natural infection and therefore may not be identified by the immunological approach (McSorley et al., 2013). Biochemical or functional analyses of parasite molecules using newly developed proteomics, secretomics, and metabolomics technologies provide new approaches to identify vaccine candidates with functions essential for the survival of parasites. Many functional proteins have been identified in the excretory/secretory products (ESPs) of *O. volvulus* that have potential as vaccine candidates (Vanhamme et al., 2020).

**Figure 2:**
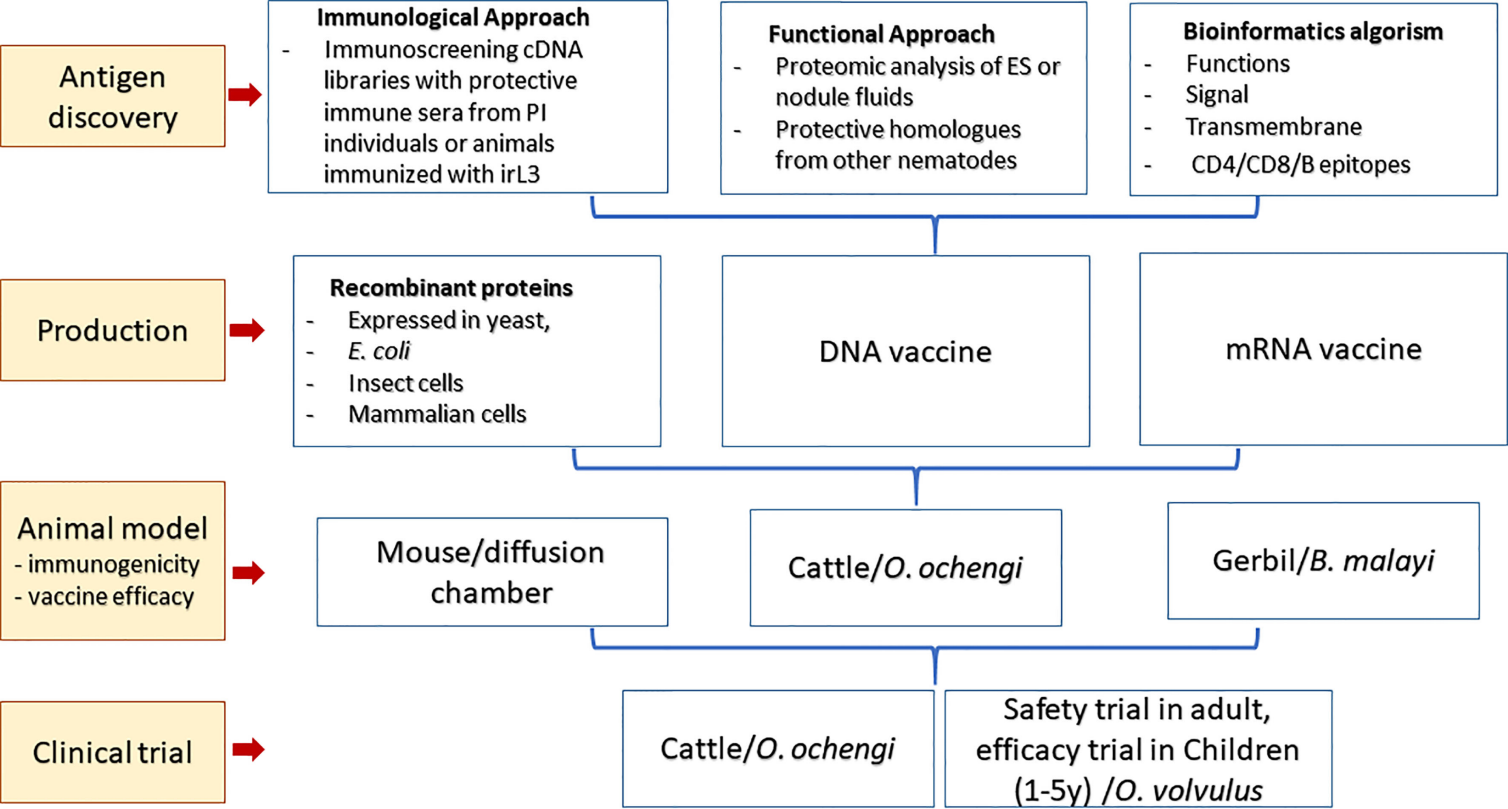
The roadmap to making vaccine against human onchocerciasis.

As more genome and transcriptome databases for *Onchocerca* spp. and other related nematodes become available, a faster and novel approach to vaccine discovery from genome analysis known as “reverse vaccinology” has been adopted to design vaccines against parasites (Kanampalliwar, 2020). By using bioinformatics tools, the whole genome sequences of a pathogen or related pathogens can be *in silico* analyzed and screened to identify DNA sequences that encode proteins bearing essential functions and vaccine properties, or immunogenic epitopes for making multi-epitope vaccines. The enormous genome sequencing information and the rise of bioinformatics have triggered a new era of vaccine research and development of the so-called “third generation” of vaccinology (de la Fuente and Contreras, 2021). Transcriptomic analyses of L3, mL3, Mf, and adult filarial stages also accelerate the identification of stage-specific antigens (Bennuru et al., 2016) and, accordingly, could identify genes essential for the early establishment of infection in the host, therefore the important targets for prophylactic vaccines of onchocerciasis (Lizotte-Waniewski et al., 2000). The overall roadmap to making vaccines against onchocerciasis is summarized in **Figure 2**.

## Lead *O. volvulus* Vaccine Candidates

Based on the observations highlighted above, two strategies are extensively used to identify antigens from *O. volvulus* with vaccine potential. One is the immunoscreening of stage-specific cDNA libraries including those critical for establishment of *O. volvulus* infection (L3 and mL3) with sera from people living in endemic areas who are putatively immune or from animals immunized with irradiated L3 and acquired protective immunity against challenge infection (Lustigman et al., 2002; Abraham et al., 2021). Another is to identify functional proteins that are essential during the infection process or maintenance of the life cycle in the host by analyzing the *O. volvulus* genome or transcriptome (Lizotte-Waniewski et al., 2000) or by studying functional homologs that induce protective immunity in other nematode host–parasite systems (Gregory et al., 2000). Some of the functional proteins were found to be present as excretory/secretory products (ESPs) in the nodule fluid or secreted by L3 during infections. Analysis of ESPs in nodule fluid in cows infected with *O. ochengi* identified 85 proteins with drug or vaccine potential (Eberle et al., 2015). Vaccination with these crucial molecules may induce immune responses that block or interfere with the establishment of the parasite in the host (Lustigman et al., 2017; Abraham et al., 2021). Another 26 protein antigens were identified through immunoscreening of a cDNA library with different protective sera, 12 of which demonstrated partial or significant protection when tested in the *O. volvulus* mouse model (Abraham et al., 2001; Lustigman et al., 2002; Lustigman et al., 2003). Following are the major identified vaccine candidates for onchocerciasis (**Table 1**).

**Table 1:** The characteristics of major vaccine candidates for onchocerciasis.

Antigen	Function(s)	Localization	Size	Immune sera used to clone	Protective evidence	Expressed host	Adjuvant	Worm reduction%	Immu effector	References
Ov-RAL-2	Novel, nematode-specific SXP/RAL-2 family	Larval/adult hypodermis; ES	17 kDa	Rabbit anti-L3, PI sera	-Recognized by PI sera -Ocular pathology -Protective homologues in Ascaris hookworm, filarial	*E. coli*	BC/FCA Alum	51%–60% 39% (Hess)	IgG1, IgG3	(Gallin et al., 1989; Bradley et al., 1993; Chakravarti et al., 1996; Lustigman et al., 2002)
						Yeast	Alum	24% (Hess)		
Ov-103	Novel, surface-associate antigen	Cuticle and hypodermis of L3, Mf	15 kDa	Chimpanzee infected sera	-Recognized by PI sera -Antibody-mediated ADCC	*E. coli*	Alum	8%		(Lustigman et al., 1992b)
						Yeast	Alum	30%	IgG1, IgG3	
Ov-ALT-1	Abundant larval transcript, secreted larval acidic protein	L3 granules esophagus	22 kDa	PI sera	-Recognized by PI -Secreted -L3-specific -Protective homologue in *B. malayi*	*E. coli*	Freund’s Alum	36% 42% combined with other 7 antigens 0%	IgG1, IgG3	(Joseph et al., 1998), (Wu et al., 2004)
OvB8	Novel, secreted	All stages, esophagus body cavity in molting L3	66 kDa	PI sera	-Recognized by PI -Involved in molting	*E. coli*	Alum	46% decrease L3 to L4 molting	Th2, IgG1	(Abraham et al., 2001)
Ov-TMY-1	Tropomyosin	Cuticle and muscle of microfilariae and L3 secreted	33 kDa	Recognized by protective immune sera	-Antibody inversely correlated with the densities of Mf -Protective homologue in *O. lienalis* and *A. viteae*	*E. coli* fused with MBP	Freund’s	48%–62%	IgG	(Jenkins et al., 1998)
Ov-CPI-2	Cysteine protease inhibitor, secreted	Hypodermis and the basal layer of the cuticle of L3 and female adult worm	17 kDa	IrL3 immune sera	-Recognized by irL3 sera and human PI -Cysteine protease inhibitor -Secreted -immunomodulatory -96%–100% inhibition of L3 to L4 molting -Protective homologues in *B. malayi* and *A. viteae*	*E. coli* and yeast)	Alum Freund’s	30% 42% combined with other 7 antigens	Th2/1 IgG-mediated ADCC	(Lustigman et al., 1992a; Cho-Ngwa et al., 2010; Hess et al., 2014)
		
Ov-B20	Novel, nematode specific	Larval hypodermic and cuticle	50 kDa	Recognized by irL3 sera	-Larval antigen -Recognized by irL3 sera -Protective homologue in *A. viteae*	*E. coli*	Freund’s	42% combined with other 7 antigens	Antibody	(Jenkins et al., 1996).
Ov-FAR-1	fatty acid and retinol binding protein secreted	Major secreted protein, located in L3 cuticle	20 kDa	Recognized by irL3 sera	-Recognized by anti-L3 and human PI -Major secreted -Retinol-binding -Homologue in *A. viteae*	*E. coli*	Freund’s	36%–55% worm reduction in jirds/A. viteae		(Jenkins et al., 1996; Kennedy et al., 1997)
Ov-CHI-1	Chitiase	L3	54 kDa	Genome	-Functional (chitinase) -Protective homologues in B. malayi, *W. bancrofti*, *A. viteae*	DNA		53%	IgG1	(Harrison et al., 1999)
Ov-ASP-1	Nematode activated secret protein	Secreted, larval abundant, esophagus glandular	15 kDa	PI sera, mice anti-Ov-L3	-Secreted -Recognized by PI -Immunogenic	*E. coli*	Freund’s Alum	42% 44%	IgG1, IgG2a	(MacDonald et al., 2004)

### Ov-CPI-2 (Ov7)

OV7 was identified by immunoscreening an adult *O. volvulus* cDNA library with chimpanzee antiserum generated against irradiated infective larvae. Western blot with antibody revealed that the native antigen of OV7 was 17 kDa located in L3, L4, and adult worms. Immunoelectron microscopy showed that OV7 was located in the hypodermis and the basal layer of the cuticle of L3 and female adult worm, and in the eggshell around developing microfilariae (Lustigman et al., 1991). Sequence alignment reveals that OV7 belongs to the cystatin superfamily of cysteine proteinase inhibitors and the recombinant OV7 protein demonstrated its activity to inhibit cysteine proteinases at physiological conditions, therefore renamed as Ov-CPI-2 (Lustigman et al., 1992a). Ov-CPI-2 is also the most abundant protein in L3 and molting L3 recognized by the sera from chronically infected human with putative immunity (Cho-Ngwa et al., 2010). Ov-CPI-2 contains a hydrophobic leader sequence, indicating its secreted extracellular function involved in the survival of the parasite in the host. The cystatin functional activity and its location suggest the role of OV7 in the regulation of parasite cysteine proteases during molting or hatching of the worms or in regulating host immune responses (Lustigman et al., 1992a). The *E. coli* expressed soluble Ov-CPI-2 protein demonstrated strong inhibitory activity on human cysteine protease cathepsins L and S (Schonemeyer et al., 2001). Further studies found that Ov-CPI-2 suppressed the antigen-driven proliferation of human PBMC, especially monocytes, through stimulating IL-10, indicating that Ov-CPI-2 is a strong immunomodulatory protein causing host cellular hyporesponsiveness, and therefore an essential factor for immune evasion and support for the survival of *O. volvulus* within its human host (Schonemeyer et al., 2001). It was further found that the molting of L3 to L4 was reduced up to 96%–100% when L3 were *in vitro* incubated with purified human anti-Ov-CPI-2 IgG in the presence of human neutrophils, indicating that Ov-CPI-2 is necessary for the molting of L3 to L4, the critical step to establishing parasitism in the terminal host. The immune response to Ov-CPI-2 may be involved in the ADCC-related mechanism in the inhibition of L3 molting (Lustigman et al., 2003; Cho-Ngwa et al., 2010). The homologues of Ov-CPI-2 were also identified in *B. malayi* (Gregory et al., 1997) and *Acanthocheilonema viteae* (Hartmann et al., 1997). However, vaccination with Bm-CPI-2 in its original form did not confer protection against *B. malayi* L3 challenge infection in gerbils but altered the location of adult worms from the lymphatic vessels to the heart and lungs (Arumugam et al., 2014b). However, vaccination with a genetically modified Bm-CPI-2 reduced adult parasite numbers and affected the fertility of female worms following a subcutaneous challenge of gerbils with *B. malayi* infective larvae (Arumugam et al., 2014b)

### Ov-RAL-2

A cDNA encoding a 17-kDa protein called Ov-RAL-2 (λRAL-2, or Ov17) was isolated from an *O. volvulus* adult cDNA expression library recognized by rabbit anti-living L3 sera. Most individuals living in the endemic areas produced antibody anti-Ov-RAL-2, indicating its strong antigenicity during natural infection (Unnasch et al., 1988; Gallin et al., 1989). Individuals with the anti-Ov-RAL-2 antibody displayed significantly less chance of developing ocular pathology associated with *O. volvulus* infection, indicating that Ov-RAL-2 may be involved with a mechanism of ocular pathology (Gallin et al., 1989). Recombinant Ov-RAL-2 expressed in *E. coli* elicited significant and sustained proliferation of lymphocytes from chimpanzees immunized with live or irradiated *O. volvulus* L3, indicating that Ov-RAL-2 is involved in the L3-induced protective immune responses (Chakravarti et al., 1996). The native Ov-RAL-2 was localized in the hypodermis of adult and larval stages of the parasite. Full-length cDNA clones encoding Ov-RAL-2 contain a polyglutamine tract immediately after a putative leader sequence. The poly-Q tract at the N-terminus is not related to the antibody response during natural infection (Bradley et al., 1993).

Ov-RAL-2 is a member of the SXP/RAL-2 protein family that exists in various nematode species containing two conserved motifs: SXP1 and SXP2. These SXP/RAL-2 family proteins include the As16 protein (Wei et al., 2017) and Asc l 5 (Ahumada et al., 2020) from *Ascaris suum*, WB14 and SXP from *Wuchereria bancrofti* (Pandiaraja et al., 2010), and allergen Ani s 5 antigen from *Anisakis simplex* (Garcia-Mayoral et al., 2014). The function of this family proteins is not clear; however, the alpha helical structures from Ani s 5 and from recombinant Ov-RAL-2 expressed in *E. coli* and yeast (unpublished) indicate their cation-binding activity involved in the interaction with host immune molecules (Garcia-Mayoral et al., 2014). The homologues of Ov-RAL-2 in *A. suum* As14 (Tsuji et al., 2001), As16 (Wei et al., 2017), and *Ancylostoma caninum* Ac16 (Fujiwara et al., 2007) displayed protective immunity when immunized as recombinant proteins against the challenge of the infective larvae. Bm-RAL-2, the Ov-RAL-2 homologue in *B. malayi* sharing 62% sequence identity, contains a DUF148 domain with unknown function by searching the Pfam database (Arumugam et al., 2016). Gerbils immunized with recombinant Bm-RAL-2 induced significant worm reduction against *B. malayi* L3 challenge (Arumugam et al., 2016).

### Ov-103

Ov-103 is a 15-kDa surface-associated antigen of *O. volvulus* identified and isolated from an adult cDNA library with serum from chimpanzee infected with *O. volvulus*. It is present in the basal layer of the cuticle and the hypodermis of microfilariae and adult female worms (Lustigman et al., 1992). It was also identified to be expressed by *O. volvulus* L3s (Lizotte-Waniewski et al., 2000). Ov-103 is an immunodominant antigen recognized by *O. volvulus*-infected human sera, especially more significantly by sera from individuals that have low levels of patent infection. In the experimental infection with *O. volvulus* infective L3, the anti-Ov-103 antibody appeared earlier in chimpanzees with low-level or non-patent infections than those with patent infection. More importantly, the anti-Ov-103 antibody could mediate the killing of nodular microfilariae in the presence of normal human granulocytes *in vitro* (Johnson et al., 1995) as well as inhibiting molting of L3 in the presence of neutrophils and monocytes (Lustigman et al., 1992, George et al., 2019). Mice immunized with recombinant Ov-103 formulated with alum induced significant protection against *O. volvulus* L3 challenge in a chamber model (Lustigman et al., 2002; Hess et al., 2014).

### OV9M

OV9M was identified and cloned by immunoscreening the *O. volvulus* adult cDNA library using pooled rabbit antisera against L3 and L4 larvae of the parasite. It is a 45-kDa immunodominant calponin-like protein located in the muscle of the different larval stages and adult worms. The antibodies to the OV9M protein are present in endemic residents with both patent and non-patent infection (Irvine et al., 1994).

### Ov-ALT-1

Immunoscreening of the *O. volvulus* L3 cDNA expression library with a pool of sera from PI individuals living in the endemic regions identified 58 cDNA clones, 6 of which encode a 20-kDa abundant larval transcript protein named Ov-ALT-1. Ov-ALT-1 is a larval stage-specific antigen belonging to a family of secreted larval acidic proteins (SLAPs), being expressed exclusively in late L2 and L3 during growth in the vector. The native Ov-ALT-1 is present in the granules of the glandular esophagus of L3 during growth in the blackfly vector (Joseph et al., 1998). It is secreted upon the resumption of development in the definitive host, therefore related to the establishment of infection in human (Wu et al., 2004). Individuals in a region of hyperendemicity in Cameroon showed age-associated IgG1 and IgG3 responses to Ov-ALT-1, indicating that immune responses to Ov-ALT-1 are associated with concomitant immunity (MacDonald et al., 2002). Mice immunized with recombinant Ov-ALT-1 produced a modest level of protection against challenge with *O. volvulus* L3 larvae (36% reduction of L4 survival rate) (Wu et al., 2004). The protective immunity induced by Ov-ALT-1 is consistent with reports of the induction of protective immunity in jirds induced by vaccination with its homologue Bm-ALT-1 in *B. malayi* (Gregory et al., 2000). The homologue of Ov-ALT-1 was also identified in the ES products of dog filarial *Dirofilaria immitis* larvae (Frank and Grieve, 1996; Frank et al., 1996).

### Ov-B20

Ov-B20 is a larval antigen of *O. volvulus* recognized by sera from cattle vaccinated with irradiated L3 of *O. lienalis*. It contains 460 amino acid residues expressed in developing stages from embryo to L4 larva, but not in the adult (Abdel-Wahab et al., 1996). The native Ov-B20 is located in the hypodermis and cuticle of infective larvae. Although vaccination of mice with B20-MBP fusion protein did not induce a reduction of microfilariae of *O. lienalis* in the ears, jirds vaccinated with B20-MBP fusion induced a 49%–60% adult worm reduction and a 97% reduction in microfilaremia against *A. viteae* infection (Taylor et al., 1995). Another *A. viteae*/Mongolian jird model trial with recombinant Ov-B20 induced a host-protective response with 36%–55% worm reduction following a challenge infection (Jenkins et al., 1996).

### Ov-TMY-1

Ov-TMY-1 is a 33-kDa tropomyosin located within muscle blocks and the cuticle of microfilariae and infective larvae. It shares 91% identity with its homologues from other nematodes. Anti-Ov-TMY-1 antibodies were abundant in protective sera of mice immunized with *O. volvulus* worm extracts, and the level of anti-Ov-TMY-1 antibodies was inversely correlated with the densities of microfilariae in the skin, indicating that Ov-TMY-1-induced antibody responses may be important in reducing worm burden (Jenkins et al., 1998). Recombinant protein containing 136 amino-acid residues from the C-terminus of Ov-TMP-1 induced significant reductions of *O. lienalis* microfilariae (48%–62%) in a mouse infection model associated with an elevated level of IgG1. Another trial with the same recombinant protein in a jirds/*A. viteae* model revealed 46% adult worm reduction compared to control groups. The anti-Ov-TMY-1 antibodies recognized the native tropomyosin in ES products released by the parasite (Taylor et al., 1996). An immunodominant B-cell epitope was mapped to the N terminus of the Ov-TMY-1 protein (AQLLAEEADRKYD) by using sera from mice with protective immunity (Jenkins et al., 1998).

### Ov-CHI-1

The cDNA coding for *O. volvulus* chitinase was cloned from *O. volvulus* (*Ov-chi-1*) with 54 kDa in size. It is expressed specifically in L3 of *O. volvulus* (Wu et al., 1996). Mice immunized with the *Ov-chi-1* DNA vaccine induced significant IgG1 production and partial protection against challenge infection with L3 larvae (53% reduction in L3 survival) in a mouse/chamber model (Harrison et al., 1999). The homologues of Ov-CHI-1 in *B. malayi* were recognized by a transmission-blocking monoclonal antibody MF1 (Fuhrman et al., 1992). A 43-kDa larval-stage chitinase of *W. bancrofti* was recognized by the sera from infection-free individuals living in an endemic area, indicating its potential protective immunity in bancroftian filariasis (Freedman et al., 1989). The homologue in *A. viteae* is believed to be involved in the degradation of the filarial cuticle during molting and thus represents a target of protective immune responses in a phase where the parasite is highly vulnerable (Adam et al., 1996). A recent study revealed that chitinase secreted by the helminth *Trichuris suis* L1 larvae mimics host chitinase and displays immunomodulatory properties in Inflammatory lung disease (Ebner et al., 2021).

### OvB8

OvB8 is a 66-kDa protein recognized by sera from PI individuals living in endemic areas in Liberia and Ecuador. It is present in all stages of the *O. volvulus* parasite, especially located in the basal lamina surrounding the esophagus and the body cavity in molting L3 (Abraham et al., 2001). There is no sequence homology to any existing nematode genes except for a homologue in the *B. malayi* EST database (Abraham et al., 2001; Lustigman et al., 2002). Immunization with recombinant OvB8 protein formulated with alum induced an up to 46% reduction in L3 survival rate and a significant decrease of molting of challenged L3 to L4 in a chamber mouse model (Abraham et al., 2001).

### Ov-FAR-1

Ov20 is an immunodominant protein recognized by antiserum raised against the infective L3 or human onchocerciasis infection sera. It is a major protein secreted by *O. volvulus* in the nodule with a molecular weight of 20 kDa. The native Ov20 was located in the L3 cuticle and uterine wall of the adult female (Jenkins et al., 1996). *E. coli* expressing recombinant Ov20 had strong binding ability to retinol and fatty acids, therefore renamed as fatty acid and retinol-binding protein of *O. volvulus* (Ov-FAR-1). The secondary structure of Ov-FAR-1 contains an alpha-helix in a coiled-coil motif determined by circular dichroism. The retinol-binding activity and the secretion property of Ov-FAR-1 indicate its potential contribution to the eye pathology associated with onchocerciasis, therefore presenting a potential target for drug or vaccine development (Kennedy et al., 1997). Indeed, a vaccine trial performed in an *A. viteae*/Mongolian jird model of filariasis displayed a 36%–55% worm reduction in jirds immunized with recombinant Ov-FAR-1 and followed with a challenge infection (Jenkins et al., 1996). In a *B*. *malayi* infection model in gerbils, immunization with r*Bm-*FAR-1 and its homolog r*Bm*-FAR-2 formulated Montanide-720 or alum elicited high titers of antigen-specific IgG, but only gerbils immunized with r*Bm*-FAR-1 formulated with Montanide-720 produced a statistically significant reduction in adult worms (68%) following challenge with *B*. *malayi* infective larvae (Zhan et al., 2018).

### Ov-FBA

A cDNA encoding the glycolytic enzyme fructose 1,6 bisphosphate aldolase of *O. volvulus* (Ov-FBA-1) was cloned by immunoscreening the *O. volvulus* cDNA library with PI sera. The native Ov-FBA is abundantly expressed in metabolically active tissues, including body wall, muscle, the reproductive tract of adult female worms, cuticle separates during molting, and the channels connecting the esophagus to the cuticle. Strong humoral and cellular immune responses to Ov-FBA-1 were observed in both PI and infected subjects. Despite the absence of immune response in parasite-exposed human populations to this antigen, the recombinant Ov-FBA-1 induced significant protective efficacy in a mouse chamber model with reduction in survival of larvae by 50% (McCarthy et al., 2002). This observation gives support for further study of this enzyme as a vaccine candidate in larger animal models.

### Ov-ASP-1

Ov-ASP-1 is an activation-associated secreted protein (ASP) belonging to a family of cysteine-rich secretory protein/antigen 5/pathogenesis related-1 proteins (CAP). It is abundantly expressed in L3 of *O. volvulus* with structural similarities to a component of vespid venom. Recombinant Ov-ASP-1 protein induced an angiogenic response after injection (Ding et al., 2018) into corneas of naive mice, suggesting its potential function involved in the eye pathology caused by the onchocerciasis (Lizotte-Waniewski et al., 2000). *O. volvulus*-infected (INF) and putatively immune (PI) individuals contain high titers of anti-Ov-ASP-1 IgG. Mice immunized with recombinant Ov-ASP-1 formulated with Freund’s or alum adjuvant produced partial but significant protection against challenge with L3 associated with high titers of Th1-related IgG2a with Freund’s adjuvant or Th2-skewed IgG1 response with alum (MacDonald et al., 2004). The protective immunity was also observed in its homologue of human hookworm *Necator americanus* (*Na*-ASP-2) (Bethony et al., 2008). Ov-ASP-1 is very immunogenic; immunization of recombinant Ov-ASP-1 without adjuvant induced high titers of both IgG1 and IgG2a in mice. Further studies revealed that Ov-ASP-1 owns strong immunostimulatory protein and exerts its adjuvanticity when combined with various vaccine antigens or commercial vaccines (MacDonald et al., 2005; He et al., 2009; Jiang et al., 2016; Jain et al., 2018).

### Multi-Epitope Vaccine

A filarial-conserved multi-epitope subunit vaccine based on 8 identified vaccine candidates was previously assessed in preclinical studies (Ov-103, Ov-RAL-2, Ov-ASP-1, Ov-ALT-1, and Ov-ALT-2, Ov-B20, Ov-RBP-1 and Ov-CHI-1) (Hess et al., 2014; Lustigman et al., 2017). This multi-epitope construct designated as Ov-DKR-2 contains B- and T-cell epitopes of these 8 immunogenic vaccine antigens. A preliminary immunological test with this multi-epitope vaccine candidate revealed its strong reaction to antibodies in sera from putatively immune or infected individuals living in *O. volvulus*-endemic areas as well as from loiasis individuals, but not with the normal control sera from European individuals. These results support its further evaluation as a vaccine candidate for onchocerciasis (Shey et al., 2021). Immuno-informatics tools and high-density peptide array were used to screen the immunodominant epitopes from the same 8 promising vaccine candidates as mentioned above using sera from onchocerciasis patients and healthy controls. The immunodominant linear epitopes were found in these proteins that were specifically recognized by onchocerciasis patient sera. This peptide-based ELISA was developed to evaluate their potential for diagnosing *Onchocerca* infection (with a sensitivity of 75.0% and a specificity of 98.5%) especially the poly-glutamine stretch of Ov-RAL-2, which could be used as a serodiagnostic marker for *O. volvulus* infection. These immunodominant epitope peptides also have potential to be developed as vaccine against onchocerciasis (Lagatie et al., 2018).

## Down-Selection of Recombinant Protein Vaccines

To down-select the antigen(s) with the most promising vaccine efficacy as vaccine candidate(s), the following criteria were used to create a priority ranking: (i) with known or essential function required in the infection and survival in the host; (ii) L3 or L4 abundant antigens responsible for the initiation of infection in host; (iii) localized on the surface area immunologically accessible to the immune responses; (iv) recognized by the antibodies from humans with putatively protective immunity (PI) or cattle, chimpanzees, and mice immunized with irradiated larvae; (v) protection identified in a mouse chamber model or the killing (or L3 molting inhibition) of larvae by the antibody in the presence of neutrophil or monocytes *in vitro*; and (vi) presence of the protective homologues in other filarial or nematodes (Lustigman et al., 2002; Lustigman et al., 2017).

Based on the selected criteria, 8 of the 12 potential vaccine candidates described above that induced worm reduction between 39% and 69% (Abraham et al., 2001; Lustigman et al., 2002; Lustigman et al., 2003; Lustigman et al., 2017) were selected for further evaluation in the mouse chamber model in parallel under the same conditions. These 8 antigens are Ov-CPI-2, Ov-ASP-1, Ov-RAL-2, Ov-ALT-1, Ov-103, Ov-B20, Ov-FBP-1, and Ov-CHI (Lustigman et al., 2017). To avoid the potential side effect of the functional cysteine protease inhibitor of Ov-CPI-2 (Gregory and Maizels, 2008) and to increase its immunogenicity in immunized hosts (Babayan et al., 2012), the Asn66 responsible for the asparaginyl endopeptidase inhibitory activity was mutated to Lys66 (Babayan et al., 2012) and thus created a mutated form of the inhibitor, Ov-CPI-1M. Seven of the eight down-selected antigens were expressed as soluble recombinant proteins in yeast *Pichia pastoris* and *E. coli* systems at Texas Children’s Hospital Center for Vaccine Development under strict conditions of quality control. Mice were immunized subcutaneously with each of the purified recombinant proteins formulated with Rehydragel™ LV (alum) for three times before being challenged with L3 in the embedded diffusion chamber. Out of seven vaccine candidates tested, Ov-103, Ov-RAL-2, and Ov-CPI-2M induced protective immunity against *O. volvulus* L3 and the protection was reproducible in separate trials (Hess et al., 2014). Mice immunized with recombinant Ov-103 expressed in yeast induced a significant 30% reduction in L3 survival and a 63% level of host protection (mice with parasite recovery lower than control mice) while recombinant Ov-103 expressed in *E. coli* did not show significant protection. Interestingly, mice immunized with *E. coli* expressed recombinant Ov-RAL-2 induced a significantly higher protection than protein expressed in yeast (L3 survival reduction 39% vs. 24%, host protection 64% vs. 55%). These protection differences between proteins expressed in *E. coli* and yeast for both Ov-103 and Ov-RAL-2 seem not to relate to the serological IgG titers and molecular structures because both Ov-103 and Ov-RAL-2 expressed in *E. coli* and yeast displayed similar and comparable α-helix structures and induced similar levels of IgG (Hess et al., 2014). However, mice immunized with Ov-CPI-2M expressed in both *E. coli* and yeast produced similar protection against *O. volvulus* L3 challenge in the diffusion chamber (30% L3 survival reduction and 17% host protection) with similar levels of antigen-specific IgG levels (Hess et al., 2014). To test for synergy of efficacy when the antigens are combined, fusion of Ov-103 and Ov-RAL-2 or Ov-103/Ov-RAL-2/Ov-CPI-2M was produced. The challenge test with the fusion revealed similar levels of protection induced by each individual protein, and there was no increased protective immunity observed in the antigen combination (Hess et al., 2014).

Confirmatory protection with adjuvanted Bm-103 and Bm-RAL-2, the *B. malayi* homologues of Ov-103 and Ov-RAL-2, was determined using the gerbil model after challenge with *B. malayi* L3 larvae subcutaneously. Gerbils immunized with yeast-expressed recombinant Bm-103 or *E. coli*-expressed recombinant Bm-RAL-2 produced 39% and 42% early worm reduction 42 days post challenge with *B. malayi* L3 and 22% or 46% reduction of adult worm burden (150 days), respectively. Vaccination with a fusion Bm-103 + Bm-RAL-2 expressed in *E. coli* or with Bm-103 and Bm-RAL-2 co-administered separately resulted in improved efficacy with consistent and significant 49%–51% and 56%–61% worm reduction at 90 days postinfection, respectively, as compared to alum adjuvant control. Immunization with both antigens (fusion or concurrent) also significantly reduced the fecundity of female worms (Arumugam et al., 2016). Both Bm-103 and Bm-RAL-2 induced elevated levels of antigen-specific IgG. The Bm-CPI-2 in its native form did not induce worm reduction but instead caused the relocation of filarial worm to the heart and lung (Arumugam et al., 2014b). However, after the functional Asn66 was mutated to Lys66, the mutant form of Bm-CPI-2, Bm-CPI-2M, significantly reduced worm burden (48.0%) as well as fertility of the female worms 90 days post challenge with *B. malayi* L3. The anti-Bm-CPI-2M sera were able to induce the killing of *B. malayi* L3s *in vitro* in the presence of gerbil peritoneal exudate cells, suggesting antibody-dependent cell-mediated cytotoxicity as a putative protective mechanism (Arumugam et al., 2014a). The results in the *B. malayi*/gerbil model further supported the selection of Ov-RAL-2, Ov-103, and Ov-CPI-2M as the most promising vaccine candidates for onchocerciasis, and Bm-103, Bm-RAL-2, and Bm-CPI-2M as the vaccine candidates for lymphatic filariasis.

## The Leading Vaccine Candidates Ov-103 and Ov-RAL-2

Ov-103 and Ob-RAL-2 are down-selected as the leading vaccine candidates for onchocerciasis for further preclinical development. Based on the down-selection criteria highlighted above, these antigens exhibit the following properties: (i) immunodominant antigens recognized by putatively immune sera (Gallin et al., 1989, Lustigman et al., 1992); (ii) larval surface-associated antigens with immunological accessibility (Gallin et al., 1989, Lustigman et al., 1992); (iii) both antigens being immunogenic, inducing strong IgG responses in immunized mice; (iv) the significantly reduced L3 survival rate in mice immunized with recombinant Ov-103 and Ov-RAL-2 alone and in combination (Hess et al., 2014); (v) antibody inducing inhibition of L3 molting (George et al., 2019) or killing of nodular microfilariae *in vitro* (Lustigman et al., 1992); and (vi) protective homologues identified in other filaria (Pandiaraja et al., 2010; Arumugam et al., 2016), *Ascaris* (Wei et al., 2017), and hookworm (Zhan et al., 2004; Fujiwara et al., 2007).

Alum-formulated recombinant Ov-103 and Ov-RAL-2 induced significantly protective immunity against L3 challenge in diffusion chambers; however, the protective level remains partial and limited. To enhance the immunological responses and improve the protective immunity, recombinant Ov-103 and Ov-RAL-2 were formulated with different adjuvants that are approved for human use and known to induce dominant Th1, Th2, or combined Th1/Th2 responses including alum, Advax 1, Advax 2, CpG oligonucleotide (CpG), and MF59. Mice immunized with different adjuvant-formulated Ov-103 or Ov-RAL-2 individually or concurrently (combination) demonstrated different Th1/Th2 immune response profiles. Without adjuvant, recombinant Ov-103 and Ov-RAL-2 themselves did not induce any protective immunity. In the adjuvant groups, only alum-, Advax 2-, and MF59-formulated Ov-103 and Ov-RAL-2 induced significant protective immunity with similar levels of larval killing and host protection. The protection was associated with Th2-biased immune responses with IgG1 as the dominant antibody (Hess et al., 2016). Co-administration of Ov-103 and Ov-RAL-2 with the three adjuvants induced similar or even higher larval killing and host protection compared to mice receiving a single antigen. The highest level of larval killing was 47% achieved in mice immunized with two antigens formulated with Advax 2 as the adjuvant, suggesting that both antigens are not immunologically competitive and confer synergistic immune responses (Abraham et al., 2021). Based on chemokine profile and levels, more neutrophils and eosinophils were involved in the protective immune response induced by Ov-103, and more macrophage and neutrophil responses were involved in Ov-RAL-2-induced immune responses (Hess et al., 2016). Both recombinant Ov-103 and Ov-RAL-2 were very immunogenic and induced high levels of antigen-specific IgG without the induction of antigen-specific IgE responses. This is important as it may prevent the induction of an allergic reaction during immunization, which has hindered the continued vaccine development for Na-ASP-2, a potential vaccine candidate for hookworm infection that failed in clinical trial due to its ability to induce IgE in vaccinated people (Diemert et al., 2012).

The role of humoral immune responses in the protective immunity induced by immunization with Ov-103 or Ov-RAL-2 was confirmed in AID^-/-^ mice that was deficient in producing IgG antibody. AID^-/-^ mice immunized with Ov-103 or Ov-RAL-2 could not produce protection against challenge of *O. volvulus* L3. Further investigation on the IgG isotypes revealed that most people who lived in endemic areas and developed with putatively immune (86%) or concomitant immunity (95%) contained anti-Ov-103 and anti-Ov-RAL-2 cytophilic antibody responses (IgG1 and IgG3). Monospecific human anti-Ov-103 and anti-Ov-RAL-2 antibodies inhibited the molting of L3 when incubated with naive human monocytes (70%–80%) and to a lesser degree in the presence of naïve human neutrophils (46% for anti-Ov-103). Moreover, the inhibition of L3 molting with anti-Ov-103 antibodies was partially dependent on contact with the monocytes, while the inhibition of molting with anti-Ov-RAL-2 antibodies required the complete contact with the monocytes, suggesting that the mechanisms by which the two antibodies cause inhibition of molting are distinct. Notably, the L3 killing in Ov-103- and Ov-RAL-2-vaccinated mice only occurred when immune cells enter the chamber where the parasites reside, suggesting that ADCC is involved in the protective immunity induced by Ov-103 and Ov-RAL-2, and balanced Th1/Th2 responses are essential for the protective immunity to *O. volvulus* (George et al., 2019).

Before being advanced to clinical vaccine trials in humans, it was important to consider whether diverse host genetics may affect vaccine efficacy. Eight different genetically diverse collaborative cross recombinant inbred intercross mouse lines (CC-RIX) were immunized with both Ov-103 and Ov-RAL-2 formulated with the adjuvant Advax 2. After challenge, significant reductions in parasite survival were observed in 7 out of 8 tested CC-RIX lines with a similar level of protection as BALB/cByJ mice. However, there were no statistically significant correlations between larval killing and specific immune cell types, either with the levels of cytokines or chemokines measured in the chamber fluid and or in culture media of *ex vivo* antigen-stimulated spleen lymphocytes. Moreover, although immunization with both Ov-103 and Ov-RAL-2 induced antigen-specific IgG1 and IgG2a/b/c in BALB/cByJ and most of CC-RIX lines with different titers, there were no statistically significant correlations between antigen-specific antibody titer and the level of larval killing. These results reveal that the Ov-103 and Ov-RAL-2 vaccines are able to induce protection against *Onchocerca* L3 infection using varied mechanisms and in a wide range of host genetic backgrounds, suggesting that the vaccines could be translated for clinical development and may protect diverse human populations from infection with *O. volvulus* (Ryan et al., 2021).

## Preclinical Trial With Bivalent Adjuvanted Ov-103 and Ov-RAL-2 Vaccine in Cattle

In 2015, an international consortium including the Texas Children’s Center for Vaccine Development and its partners in US, Europe, and Africa launched The Onchocerciasis Vaccine for Africa Initiative (TOVA) with the goal of developing and evaluating an onchocerciasis vaccine for Africa (Hotez et al., 2015). Intense laboratory down-selection led to the selection of two antigens, Ov-103 and Ov-RAL-2, for further clinical development based on their consistent protective efficacies in mouse model studies. Although recombinant Ov-CPI-2M with functional Asn66 mutated to Lys66 induced a similar level of protection against *O. volvulus* L3 challenge, it was down-selected because of its ability to induce IgE responses in children ≤5 years of age. In comparison, there were no IgE responses found in Ov-RAL-2 and Ov-103 immunization (Abraham et al., 2021).

Currently, TOVA is moving forward to a preclinical trial in Cameroon for the immunogenicity and vaccine efficacy of co-administered Ov-RAL-2 and Ov-103 in cattle exposed to natural infection of *O. ochengi* (Makepeace et al., 2015). Due to the similarity of the life cycle transmitted by the same fly vector, overlapped endemic regions, and highly related vaccine candidates between *O. volvulus* in human and *O. ochengi* in cattle, the natural infection of cattle with *O. ochengi* in endemic areas is selected as the only one field vaccine trial model for validating the vaccine efficacy for human onchocerciasis (Makepeace and Tanya, 2016). Vaccination against onchocerciasis in natural host–parasite relationships represents the real-world vaccine efficacy in endemic regions. The data from the cattle trial in endemic regions will be translated to a phase I clinical trial in human. The target product profiles (TPPs) for developing an onchocerciasis vaccine for African living in endemic areas include the co-administration of Advax-2-adjuvanted Ov-103 and Ov-RAL-2 in children 1–5 years old (Hotez et al., 2015). However, a safety trial will be done in adults living in non-endemic and then in endemic regions to ensure that the vaccine is safe enough before it is tested in children. The aim of this vaccine is to prevent the establishment of infection in children before exposure to the infection. A serological investigation found that all 73 children 1–5 years old from a highly endemic region in Ghana did not show any anti-Ov-103 and Ov-RAL-2 IgE responses, while 3/27 and 1/27 of the children 6–8 years old displayed anti-Ov-103 or Ov-RAL-2 IgE responses, suggesting that continuous exposure to infective larvae in children over 5 years of age may develop IgE response to native Ov-103 and/or Ov-RAL-2 proteins during infection (Abraham et al., 2021). Therefore, the Ov-103 and Ov-RAL-2 vaccines should be safe for targeting children under 5 years of age without concern of pathological atopic responses as was other helminth vaccines when tested in humans (Hamilton et al., 2010; Diemert et al., 2012).

## Conclusions and Perspectives

After decades of unremitting efforts by researchers around the world, especially by the close collaboration among the New York Blood Center (Dr. Sara Lustigman), Thomas Jefferson University (Dr. David Abraham), and Texas Children’s Hospital Center for Vaccine Development (Dr. Peter Hotez and Maria Elena Bottazzi), two antigens Ov-103 and Ov-RAL-2 have been selected for a bivalent vaccine for clinical efficacy trials based on their consistent protective efficacy induced in mice. This bivalent vaccine has been moved forward to a clinical trial in naturally infected cattle in Cameroon with the exciting endorsement of TOVA. Efficacy results from the cattle clinical trial with adjuvanted bivalent Ov-103 and Ov-RAL-2 vaccines will provide critical information for their immunogenicity and protective efficacy on worm burden and patency under natural infection conditions. If their protective efficacy is confirmed in the cattle clinical trial, the first bivalent onchocerciasis vaccine with Advax-2- or alum-formulated bivalent Ov-103 and Ov-RAL-2 will be produced in large scale under cGMP conditions and used to perform a phase I safety clinical trial. The ultimate goal is to develop a vaccine that can be used safely in children under 5 years of age in endemic regions that prevents infection with *O. volvulus* and protects them from developing adult worm pathological consequences with time.

While we are testing our first generation of the onchocerciasis vaccine, the co-administration of recombinant Ov-103 and Ov-RAL-2 adjuvanted with Montanide™ ISA 206VG in cattle in a preclinical trial in Africa, there still are some challenges. Notably, the protection rate of current selected Ov-103 and Ov-RAL-2 against *Onchocerca* L3 challenge is partial and limited despite the intensive search for the appropriate adjuvants and immunization regime to induce better protection. We will need to continue modeling whether the partial protection rate of current selected Ov-103 and Ov-RAL-2 against *Onchocerca* L3 challenge will be sufficient to support elimination (Turner et al., 2015). We also need to make more efforts to enhance their immunogenicity and vaccine efficacy by (i) searching and identifying the optimal adjuvant and formulation that might induce better protective immunity in humans; (ii) optimizing the co-administration of both antigens by making a fusion protein or combination of both individual antigens, to reach better protective efficacy; (iii) increasing efforts for the discovery of additional effective vaccine candidates, including new-generation vaccine antigens identified using new technologies such as functional genomics, transcriptomic, proteomic, secretomics, immunomics, and the mRNA vaccine technology; (iv) developing novel diagnostic assays that can support the rapid monitoring of vaccine efficacy in human clinical trials; (v) developing suitable product development strategies and the preferred target product profile; (vi) enhancing the collaboration between laboratory researchers and local health workers; (vii) promoting financial support from international organizations and/or governments to bring such a vaccine toward clinical trial and licensure; and (viii) advocating integration of such a vaccine with other elimination programs, including South-Sahara Africa government agencies in such efforts.

## Author Contributions

BZ conceived and drafted the manuscript. SL, PH, and MB revised and added insight into the manuscript. All authors contributed to the article and approved the submitted version.

## Funding

This work was supported by NIH/NIAID R01AI078314.

## Conflict of Interest

The authors declare that the research was conducted in the absence of any commercial or financial relationships that could be construed as a potential conflict of interest.

## Publisher’s Note

All claims expressed in this article are solely those of the authors and do not necessarily represent those of their affiliated organizations, or those of the publisher, the editors and the reviewers. Any product that may be evaluated in this article, or claim that may be made by its manufacturer, is not guaranteed or endorsed by the publisher.
